# What is the Role of Point of Care Ultrasound for Suspected Pulled Elbow in Children? A Narrative Literature Review.

**DOI:** 10.24908/pocusj.v10i01.17853

**Published:** 2025-04-15

**Authors:** Salmah Lashhab, David J. McCreary

**Affiliations:** 1 Paediatric Emergency Medicine Department, Sunderland Royal Hospital, South Tyneside and Sunderland NHS Foundation Trust, Sunderland, GBR

**Keywords:** point of care ultrasound, pulled elbow, diagnosis, management

## Abstract

**Objective:**

Our objective was to evaluate and appraise the existing evidence on the use of point of care ultrasound (POCUS) for pulled elbow, including its positive findings and their reliability.

**Methods:**

We searched PubMed, Medline, EMBASE, CINAHL and Google Scholar for prospective and retrospective studies evaluating POCUS use for suspected pulled elbow. We identified positive sonographic findings along with their sensitivity and specificity relating to this diagnosis.

**Results:**

We included 13 studies that reviewed ultrasonographic findings in suspected pulled elbow. These studies discussed a range of sonographic findings between them, including radio- capitellar distance, ‘J-sign'/‘Hook sign', fat pad sign and partial eclipse sign. The studies were of mixed quality and were susceptible to bias.

**Conclusions:**

Children presenting with suspected pulled elbow who have evidence of hook sign (or J-sign) and an absence of elbow effusion on POCUS can be diagnosed with pulled elbow and safely undergo reduction. POCUS can be used following reduction to demonstrate resolution of these signs and confirm its success. Elbow injuries with effusion are likely to have bony injury, meaning that X-ray is required. Additional prospective study of children presenting with elbow injury would be required to accurately determine the effectiveness of POCUS in the diagnosis of pulled elbow.

## Background

Pulled elbow, also known as “nursemaid's elbow” or “radial head subluxation,” is a common injury presenting to the paediatric emergency department in young children, typically between the ages of one and four-years [1]. It is caused by longitudinal traction from a sudden pull of the extended and pronated arm, often from a caregiver or sibling when attempting to prevent a fall or pull a child back from danger. This leads to a subluxation of the radial head from beneath the annular ligament which holds it in place. Children typically present with a reluctance to move the affected arm following a typical history, although in up to 50% of cases the history is atypical or lacking altogether [2]. Diagnosis has traditionally been a clinical one. Pulled elbow can be confirmed by the child's immediate improvement following manipulative reduction, heralded by the satisfying ‘click' that is heard and felt when performed by the clinician. However, examination can be difficult in an upset or frightened child, and as such the precise location of the injury is not always easily identifiable. As a result, many children undergo three upper limb X-rays (as high as 28% in one study)—many of which are not necessary [3]. More concerningly, some children undergo attempted reduction for suspected pulled elbow when, in fact, a fracture is the cause of their pain [4].

Efforts have been made to identify diagnostic findings on radiographs for pulled elbow, however, this has not been demonstrated as a reliable tool [5]. To those who utilize it, POCUS is an effective diagnostic tool for undifferentiated elbow injuries. POCUS allows for immediate, real-time views of the anatomy and is both non-radiating and well-tolerated by children [5].

## Objective

This literature review aimed to evaluate and appraise the existing evidence on positive ultrasound findings for pulled elbow, the use of POCUS for its identification, and the use of POCUS confirming its successful reduction.

## Methods

**Figure 1.**
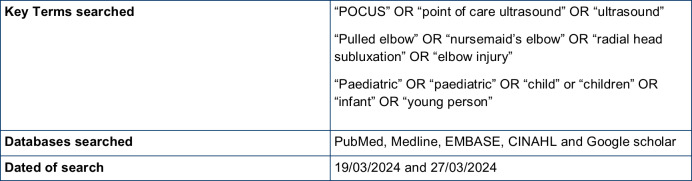
Search strategy (TIFF)

**Figure 2.**
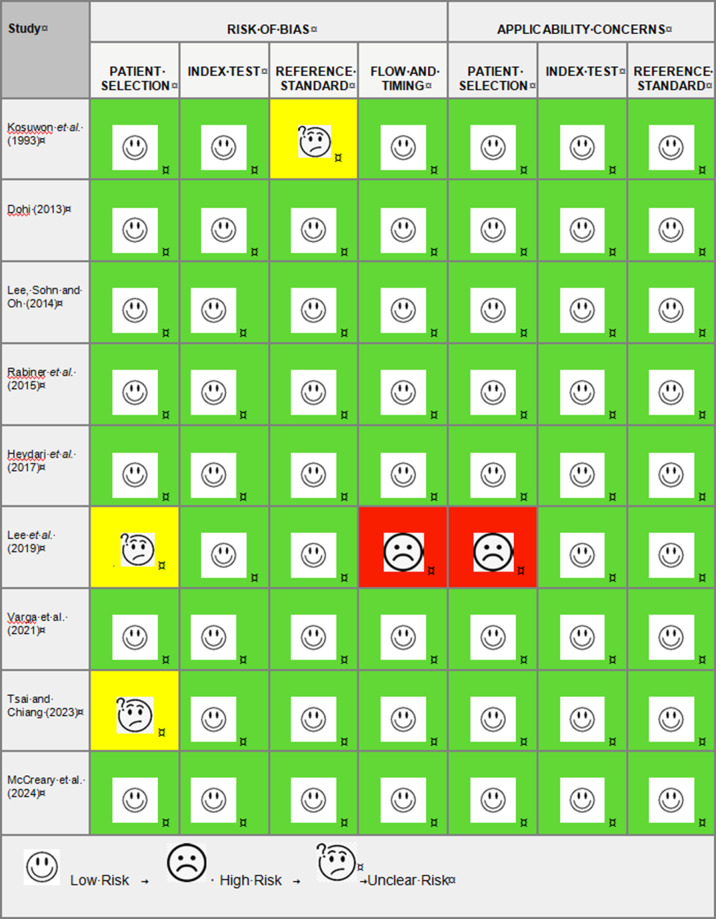
Table summarizing risk of bias QUADAS-2

Articles were sourced from PubMed, Medline, EMBASE, CINAHL and Google Scholar; see Figure 1 for search criteria. Studies were eligible for inclusion if they involved patients presenting with suspected pulled elbow and had POCUS performed. Each study was examined in relation to its description of the findings on POCUS and the reporting criteria such as diagnostic accuracy or reliability. The QUADAS-2 framework was used to appraise the studies for relevance and risk of bias and is summarized in Figure 2.

Data was extracted on the following: author and year of publication, location of study, study type, patient characteristics (including age ranges and mean age as available), sample size, and the individual performing the POCUS. All articles were independently screened by each author for suitability, with inclusion decisions reached by consensus.

## Results

**Figure 3.**
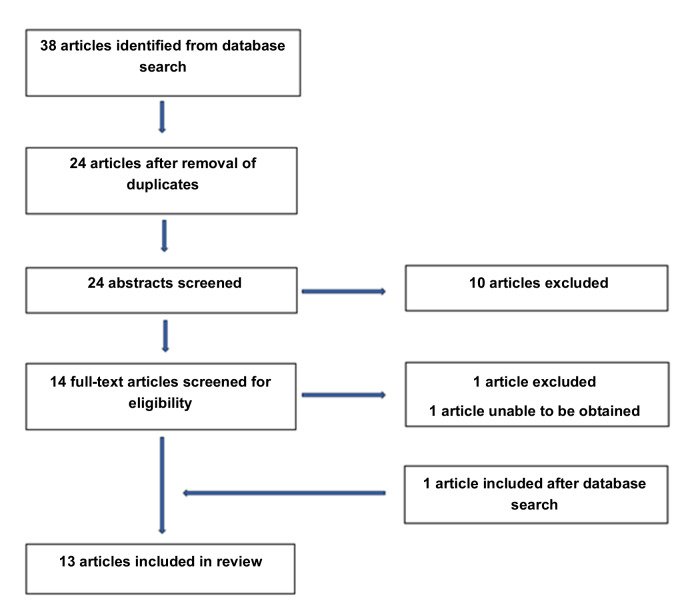
Article Selection process

A total of 38 articles were found based on database searches. After duplicates were removed, 24 articles remained. After reviewing abstracts, the full texts of 14 articles were obtained and screened for relevance to the study question. The full text for one study could not be obtained for review. One article was removed as it related to the classification of pulled elbow based on ultrasound findings rather than use of POCUS for identification of pulled elbow. One study was included after the initial searches. This left 13 studies for final analysis; See Figure 3 for a flowchart of the selection process.

There was some variation in how patients were assessed using POCUS based on anatomical positioning. For instance, assessment varied based on flexion/extension, pronation/supination, and the plane of insonation with the ultrasound probe (longitudinal or transverse), with images obtained in both the dorsal and ventral planes. Findings are summarized in Table 1. Studies were mixed in result analysis, as summarized in Table 2.

**Table 1.** Summary of included studies (continued on next page)

Study	Location	Study type	Patient characteristics	Sample Size	Ultrasound/point of care ultrasound (POCUS) performed by	Purpose	Ultrasound findings	Conclusion
**Kosuwon *et al*. (1993)**	Khon Kaen, Thailand	Case-control study	2-5 years, 14 male and 6 female	20	Unknown	The use of ultrasound for documenting pulled elbow in children	Radio-capitellar distance increase	Ultrasound can be used to document and confirm pulled elbow
**Shabat *et al*. (2005)**	Tel Aviv, Israel	Case report	4 years	1	Unknown	Confirmation of pulled elbow by ultrasound	Displacement of the cartilaginous head of the radius away from the capitellum	Ultrasound can be used to confirm pulled elbow in the uncertain diagnosis
**Dohi (2013)**	Higashi Hiroshima, Japan	Retrospective diagnostic study	4 months to 6 years	70	Unknown	To identify pulled elbow signs on ultrasound	J-sign, disappearance post-reduction	Ultrasound can be used to both diagnose pulled elbow and confirm successful reduction
**Youdong *et al*. (2014)**	Gangwon-do, Republic of Korea	Case series	6 months and 4 years	2	Unknown	Identification of hook sign in pulled elbow	Hook sign	Hook sign identifies pulled elbow which can be most useful in cases of failed reduction
**Lee, Sohn and Oh (2014)**	Anyang, Korea	Retrospective study	28.5±12.3 months	141 total, 78 included	Two emergency residents	Review ultrasound accuracy for a diagnosis of pulled elbow	Displaced annular ligament	Ultrasound can offer a preferred technique to diagnose pulled elbow than other methods
**Rabiner *et al*. (2015)**	New York, United States of America	Prospective study	Mean age of 22.3 months	42	Paediatric emergency physicians/ fellows	Presence of an elevated posterior fat pad	Posterior fat pad Lipohaemarthrosis	Elevated PFP and lipohaemarthrosis are possible findings in pulled elbow Negative ultrasound may allow for elbow to be reduced
**Güngör and Kılıç (2017)**	Antalya, Turkey	Case series	7 months, 18 months and 3 years	3	Two emergency physicians	POCUS can assist in decision-making and clinicalmanagement for patients in atypical cases of pulled elbow	Hook sign	Ultrasound can be used in diagnosing pulled elbow in cases with unknown mechanism of injury or atypical histories and also be used to assess successful reduction
**Heydari *et al*. (2017)**	Isfahan, Iran	Cross-sectional study	4 months to 6 years (mean 2 years 7 months)	60	One physician, unspecified	Use of ultrasound to diagnose and confirm successful reduction	J signSensitivity 89.1% specificity 100% in confirming treatment of pulled elbow	The sensitivity and specificity of ultrasound in confirming the considered therapeutic method for the treatment of pulled elbow was obtained higher than 90%
**Lee *et al*. (2019)**	Seoul, Korea	Retrospective study	1.25–9.5 years (mean 4.34 years)	37	Paediatric orthopaedic surgeon	To assess the use of ultrasound in the atypical pulled elbow	J-sign present 100%Coronoid and olecranon fossa effusion 100%Post-reduction ultrasound 100% had both a restored annular ligament and disentangled and swollen supinator	In atypical circumstances, ultrasound can be used for detecting an entrapped supinator and confirming adequate reduction via restoration of the annular ligament in children
**Varga et al. (2021)**	Budapest, Hungary	Prospective study	0–5 years (mean 2.3 years)	205	Orthopaedic surgeons Orthopaedic resident	If two-plane POCUS can aid in the diagnosis of pulled elbow, as well as rule out fractures	Synovial fringe enlargement (SFE) To diagnose pulled elbow, SFE sensitivity 79.5%, specificity 100% Positive fat pad with no SFE 100% sensitive and specific for fracture diagnosis	Sonography is effective method in the differential diagnosis of pulled elbow Suggest the use of ultrasound routinely before any reduction attempts in all pulled elbow cases
**McCreary and White (2023)**	Sunderland, United Kingdom	Case report	2 years	1	Emergency department physician	Incorporating POCUS as a means of increasing the reliability of findings on clinical examination	Hook sign Prominent synovial fringe	X-rays have no role in confirming nursemaid's elbow or resolution following manipulation POCUS can be used to confirm that successful reduction
**Tsai and Chiang (2023)**	Taipei City, Taiwan	Prospective Case Series	Unknown	13	1 Orthopaedic surgeon	To determine the aetiology and possible pathophysiology of pulled elbow	Partial eclipse sign 100% patients demonstrated and resolved post-reduction Synovial fringe impingement	Ultrasound can be used to diagnose pulled elbow and prevent unnecessary reduction in cases that mimic pulled elbow
**McCreary et al. (2024)**	Sunderland, United Kingdom	Retrospective case series	0-5 years	37	2 emergency consultants with POCUS qualifications	Identification of the hook sign as a positive sonographic finding for pulled elbow	Hook sign 100% sensitivity and specificity	POCUS as part of clinical assessment is effective at diagnosing pulled elbow

**Table 2.** Study analysis

Finding	Study	Sensitvity/specificity
**Radio-capitellar distance**	*Kosuwon et al. (1993)*	Not reported
	*Shabat et al. (2005)*	Not applicable
**J sign/Hook sign**	*Dohi (2013)*	Sensitivity 100% Specificity 100%
	*Youdong et al. (2014)*	Not reported in study.
	*Lee, Sohn and Oh (2014)*	Sensitivity 64.9% Specificity 100%
	*Güngör and Kılıç (2017)*	Not applicable
	*Heydari et al. (2017)*	Sensitivity 89.1% Specificity 100%
	*McCreary and White (2023)*	Not applicable
	*Lee et al. (2019)*	Not applicable
	*McCreary, Tambe and Mullen (2024)*	Sensitivity 100% Specificity 100%
	*Varga et al. (2021)*	Sensitivity 79.5% Specificity 100%
**Fat pad sign**	*Rabiner et al. (2015)*	Not reported
	*Varga et al. (2021)*	(With no SFE) Sensitivity 100% and specificity 100% for fracture diagnosis
**Partial eclipse sign**	*Tsai and Chiang (2023)*	Not reported

### Radio-capitellar Distance

The earliest documentation of ultrasound for diagnosing pulled elbow was in 1993 by Kosuwon et al. [6]. This study included 20 patients, half of whom were diagnosed with pulled elbow and the other half had no injury. Each group had 7 boys and 3 girls, with ages ranging between 2-5-years-old. Pulled elbow was confirmed based on history, examination and successful reduction. The radio-capitellar distance (RCD) was measured with the elbow flexed and both the affected and unaffected elbows scanned. The elbow was examined in pronation before manipulation and in supination post-manipulation. Results demonstrated an increased RCD of approximately 3 mm. Blind measurements of the RCD were also taken. Kosuwon et al. [6] concluded that the RCD increases and therefore ultrasound can be used to document and confirm pulled elbow. The sample size in this study was limited. Although there was evidence of a discrepancy of RCD distance between the healthy subject and the affected subject, this lacked sensitivity and specificity for a diagnosis of pulled elbow and so must be considered with caution. Furthermore, using a small measurement can be subject to error with no cutoff point regarding normal values.

Shabat et al. [7] described a case report of a 4-year-old presenting to the emergency department at Sapir Medical Center in Tel Aviv, Israel, after falling onto her extended right elbow. Initial X-ray suggested possible pulled elbow. Ultrasound of both the affected and unaffected elbows were performed. Longitudinal views of the lateral aspect of the extended elbow showed displacement of the cartilaginous head of the radius away from the capitellum in the affected elbow (compared with the normal elbow) which diagnosed pulled elbow. Shabat et al. [7] therefore recommended ultrasound to confirm pulled elbow in the uncertain diagnosis. It is unclear what the experience level of the person performing the ultrasound examination was. It is important to note that this recommendation was based on a single patient.

### J-sign/Hook sign

The ‘J-signߣ is described as the entrapment of the supinator muscle and annular ligament in the radio-humeral joint. As discussed by Dohi [8], this sign was observed by Minagawa on a review of ultrasound images obtained from 32 children diagnosed with pulled elbow. These images were achieved in longitudinal view of the anterior surface of the radio-humeral joint. The study itself could not be obtained and therefore its reliability cannot be commented on, but it has been documented in other studies evaluating ultrasound use in pulled elbow.

Dohi subsequently reviewed 70 cases of pulled elbow seen in 2010-2013 at Dohi Orthopaedic Hospital, Higashi Hiroshima, Japan. Ages ranged from 4 months to 6-years. A total of 11 patients presented with an unknown mechanism of injury. The children had both the affected and unaffected limbs scanned for comparison, with the affected limb re-scanned post-reduction. POCUS was performed in longitudinal view of radio-humeral joint (anterior surface). In the unaffected elbow, POCUS showed a normal annular ligament. The study observed both the annular ligament and supinator muscle trapped in the radio- humeral joint forming a hypoechoic J-shape in all cases, and the disappearance of the J-sign post-reduction. No audible click was heard on reduction in 10 patients, however, POCUS demonstrated successful reduction. Dohi [8] reported that the sensitivity, specificity, and the accuracy of POCUS diagnosis of the J-shaped hypoechoic image were each 100%.

Based on these findings, Dohi recommended that POCUS can be used to both diagnose pulled elbow and to confirm successful reduction through visualization and seeing subsequent disappearance of the J-sign. Limitations to this study included the small sample size. It was also a retrospective study and therefore prone to bias. Furthermore, there was no record of who performed the POCUS scans, their clinical background or inter-observer agreement between them.

Youdong et al. [9] detailed two case studies of children presenting with pulled elbow at the Department of Emergency Medicine in Gangwon-do, Republic of Korea. These patients, ages 6 months and 4-years respectively, were scanned both pre- and post-reduction. They reported ‘blunting' and ‘trapping' of the supinator muscle within the joint space, coining the term ‘hook sign' based on the ultrasonographic appearance this makes. With the elbow reduced, the supinator muscle returns to its usual position above the radial head. Again, it was not reported who performed POCUS and their clinical background or any inter-observer agreement. Their discussion highlighted the variability in operator skill.

Lee, Sohn & Oh [10] performed a retrospective study of 141 patients with suspected pulled elbow, encompassing relevant history and clinical signs and excluding those with the presence of bony crepitus, deformity, focal edema, or discoloration. Patients were determined to have a pulled elbow if they could flex and rotate the affected arm without pain post-reduction. Of the 78 patients that were included, 43 patients had a POCUS examination. The mean age was 28.5±12.3 months. Both affected and unaffected elbows were scanned, with the elbow extended and the anterior portion of the radio-capitellar joint longitudinally visualized. Three findings were documented, including (1) supinator muscle shape and if there was a change, (2) annular ligament placement, and (3) evidence of synovial fringe enlargement (SFE). The above findings in the affected versus unaffected arm were deemed significantly different with McNemar tests.

‘Loss of annular ligament in place' was reported as the only statistically significant finding (p<0.001). The sensitivity and specificity were 64.9% and 100%, respectively. Based on these findings, the study concluded that ultrasound can offer a preferred technique to diagnose pulled elbow than other methods, by way of identifying a displaced annular ligament [10]. This was a relatively small study population size. POCUS scanning was performed by two emergency residents who were given a 1-hour training and a 1-hour hands-on practice.

These images were then viewed by an expert physician who was blinded. As a retrospective study, this study may be susceptible to selection bias. As such, the authors concluded that a randomized controlled prospective study is required.

Güngör and Kılıç [11] presented three case studies within the Emergency Medicine Department, Antalya Training and Research Hospital, Antalya, Turkey. The ages for each case were 7 months, 18 months, and 3-years, respectively. POCUS was carried out by two emergency physicians with at least three years of experience who collectively reviewed the images and correlated the findings. The patient was positioned with an extended elbow, and longitudinal views in the dorsal aspect of the elbow were obtained, visualizing the radio- capitellar joint. All cases demonstrated presence of the hook sign. One case had an unclear mechanism of injury, however, with the hook sign present on POCUS examination, the elbow was reduced. No click was audible but the post-reduction images showed resolution of the hook sign, which indicated a successful reduction. Güngör and Kılıç [11] concluded that POCUS can be used to diagnose pulled elbow in cases with unknown mechanisms of injury or atypical histories. Based on these case studies it could also be used to assess successful reduction. Both physicians scanning had experience in POCUS and all images were reviewed between them to confirm findings.

Heydari et al. [12] performed a cross-sectional study between 2014-2015 at the Emergency Medicine Department, Alzahra Hospital, Medical University of Isfahan, Isfahan, Iran. A total of 60 patients were included, all diagnosed with pulled elbow, who consented to participate. The objective was to use POCUS to diagnose and confirm successful reduction. Ages ranged between 4 months to 6-years (mean 2 years and 7 months). Patients were scanned in extension, with affected and unaffected limbs assessed both pre- and post-reduction in the longitudinal plane with an anterior view of the radio-humeral joint. Pulled elbow was confirmed on POCUS with a J-shape view in the radio-humeral joint. Post-reduction, 49 patients had normal POCUS, whereas 11 were abnormal. Later, five patients with abnormal POCUS were identified with fractures and referred to the orthopaedic team. The six patients with abnormal POCUS were deemed to have had unsuccessful and unreliable scans due to either obesity or excessive weight loss. Therefore, they were observed in the emergency department. Four of these patients had a further examination and were discharged, whereas two patients had X-rays of the affected limb due to an inability to examine and were subsequently discharged. The sensitivity and specificity of ultrasound in confirming the treatment of pulled elbow were 89.1% and 100%, respectively. The accuracy of POCUS in confirming the treatment of pulled elbow was reported as 92%. This accuracy is calculated as the sum of true positives and true negatives divided by the total number of tests conducted. All patients were considered to have pulled elbow with visualization of the J-sign. This study was limited and lacked power due to its sample size. One ultrasound machine was used for all scans to reduce test error and a single physician performed all scans, although their background was unclear. There was also no reference as to what constituted an abnormal POCUS which, given this category had final diagnoses of both pulled elbow and fractures, would have helped to determine if abnormal findings were prevalent in each diagnosis.

McCreary and White [5] documented a case of a 2-year-old presenting to the Paediatric Emergency Department, Sunderland Royal Hospital, with an undifferentiated arm injury.

**Figure 4.**
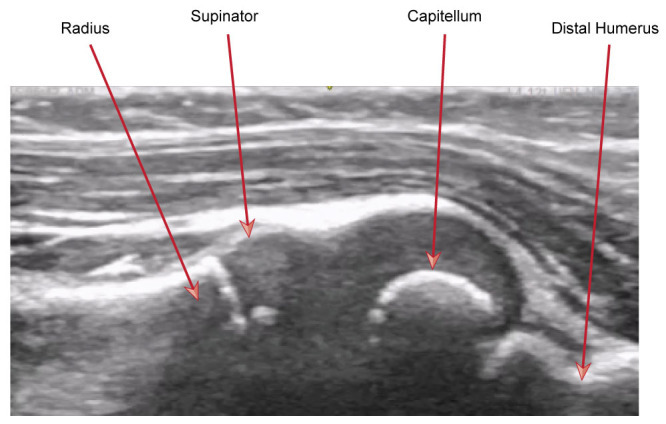
From :D. McCreary and A. White (2023). Use of POCUS for the Paediatric Patient with an Undifferentiated Upper Limb Injury. DOI: 10.24908/pocus.v8i1.15867. POCUS Journal [5, p36]. Longitudinal view of radial head demonstrating normal anatomy.

**Figure 5.**
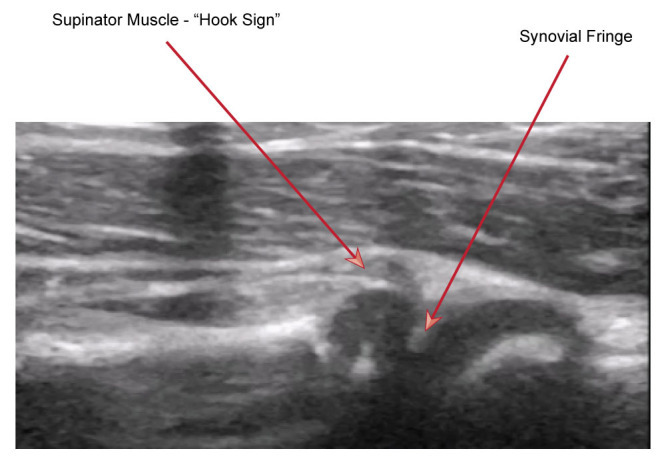
From :D. McCreary and A. White (2023). Use of POCUS for the Paediatric Patient with an Undifferentiated Upper Limb Injury. DOI: 10.24908/pocus.v8i1.15867. POCUS Journal [5: p37]. Longitudinal view of radial head demonstrating ‘hook/J sign'.

The child was distressed and holding her arm in adduction. Given the unknown history of the injury, POCUS was used to assess for both bony injury and pulled elbow, with visualization along the radio-capitellar line to assess for pulled elbow. POCUS showed ‘curling' of the supinator muscle over the radial head, referred to in this report as the hook sign. This was compared with the unaffected limb. This case report suggests two signs present on ultrasound, the first being the hook sign, and the second, a more prominent synovial fringe due to it dipping into the joint space with a more ‘pointed' appearance; see Figures 4 and 5.

Lee et al. [13] carried out a retrospective study of 37 patients with pulled elbow who had either an atypical history or failed reduction. The study, conducted at the Samsung Medical Centre, a tertiary hospital in Seoul, Korea, reviewed cases from April 2015 to September 2018. Its aim was to evaluate X-ray findings and assess the use of POCUS in atypical pulled elbow cases. The medical records and images of these patients were reviewed; the age range was between 1.25–9.5-years (mean = 4.34-years). All POCUS examinations were performed by a paediatric orthopaedic surgeon, with the elbow extended and with an anterior approach in the longitudinal view to visualize the radio-capitellar joint. Both the affected and non-affected sides were scanned. Of the 37 patients, all affected elbows demonstrated an entrapped supinator on POCUS (J-sign). Coronoid and olecranon fossa effusion was noted in 100% of cases. Absent annular ligament was present in 74% of cases on POCUS and 63% showed a deep synovial fringe. Posterior fat pad (PFP) signs were present on X-ray in 16% of cases. Post-reduction POCUS demonstrated that 100% of cases had both a restored annular ligament and a disentangled and swollen supinator. Supinator effusion was demonstrated in 58% of cases, and a retrieved synovial fringe on POCUS scan also demonstrated in 58% of cases. The supinator muscle returned to baseline on POCUS after reduction of the elbow [13]. This finding was consistent with the results of both Dohi and Minagawa as cited by Dohi, [8] suggesting the J-sign (indicative of an entrapped supinator muscle) is highly diagnostic of pulled elbow, and a resolution of this sign demonstrates successful reduction. However, this study had a small sample size and was a retrospective study, so was prone to bias. The tertiary hospital where this study was conducted received more serious injuries compared with other hospitals. Therefore, the cohort does not necessarily represent the general population presenting with pulled elbow, such as those seen in paediatric emergency departments. This is because patients in this study had all been referred to the orthopaedic team for a second opinion.

The control in this case was the non-affected elbow of the children with pulled elbow, rather than subjects from a healthy population (i.e., with normal unaffected limb). Given this, it was inconclusive whether this cohort had a level of variation in anatomical findings of the supinator muscle, synovial fringe or the degree of effusion. Bias was reduced by using the same experienced surgeon to carry out the POCUS scans, aiming to reduce inexperience bias and to reduce inter-observer error. However, clinical background regarding ultrasound training was not referenced.

McCreary, Tambe and Mullen [14] published a single-centre retrospective case series in which they examined elbow injuries for patients aged 0-5-years presenting over a 20-year period to the paediatric emergency department of the District General Hospital in the United Kingdom. This study used identification of the hook sign as a positive sonographic finding for pulled elbow.

Patients were excluded if they had obvious deformity, significant swelling or if they had a major trauma mechanism of injury. POCUS was performed by two consultants with postgraduate qualifications in POCUS according to standardized departmental protocols. A total of 110 patients were eligible for inclusion of which 37 had underwent POCUS as part of their assessment. Of these, 29 had a typical history and all had a positive POCUS for pulled elbow. Of the remaining eight patients with an atypical history, three were found to have a positive POCUS compared to unaffected elbow. The five patients with an atypical history had negative POCUS findings and were diagnosed as soft tissue injuries after further assessment. Their specificity was 100% (47.8-100) (p = 0.00003) and sensitivity was 100%. They concluded that POCUS as part of clinical assessment is very effective at diagnosing pulled elbow, especially when an atypical history is provided. Limitations of this case series included its retrospective nature and the fact that scans were only carried out when POCUS-trained consultants were present in the paediatric emergency department. As a result, the overall sample size was limited and selection bias was introduced.

### Fat Pad Sign

As discussed by Lee et al. [13], there is evidence suggesting the presence of fat pad sign on imaging of pulled elbow. Rabiner et al. [15] performed a prospective study between January 2011 and May 2012, in a paediatric emergency department in New York, to look for the presence of an elevated PFP on ultrasound with pulled elbow. Based on history and examination, 42 patients were diagnosed with pulled elbow and enrolled in the study, with a mean age of 22.3 months. POCUS examinations of both the affected and unaffected elbows were performed with the elbow at 90 degrees flexion from the dorsal aspect. Both longitudinal and transverse views were obtained. All patients were confirmed to have pulled elbow. No PFP elevation was demonstrated in 35 patients. However, 6 patients (14%) had an elevated fat pad, and 2 lipohaemarthrosis. One patient had both PFP and lipohaemarthrosis.

Reduction was successful in 100% of patients (defined as return to normal movement in the affected limb), and there were no complications reported on follow-up. The study concluded that both elevated PFP and lipohaemarthrosis are possible findings in pulled elbow but a negative ultrasound may allow for the elbow to be reduced. This study supports the presence of fat pad sign in pulled elbow. In this study, POCUS was carried out by a group of paediatric emergency physicians/fellows. This group attended a one-hour teaching session on identifying the presence of PFP. Most within the group were not highly skilled in POCUS. Post-study image review suggested that PFPs identified by the emergency clinicians were minimally elevated when compared with PFPs related to fractures which are usually more marked. Given the prospective nature of this study with enrolment of patients only when a trained clinician was available, the sample size was small. Uncertain diagnoses or patients who had X-rays performed were excluded from the study.

Varga et al. [16] performed a prospective diagnostic study to assess if POCUS, specifically a two-plane POCUS examination, can aid in the diagnosis of pulled elbow, as well as rule out fractures. Between 2016 and 2017, 205 patients (mean age 2.3-years) were included who were clinically diagnosed with pulled elbow. The patient's elbow was extended, and longitudinal views were captured in both the dorsal-plane of the olecranon fossa and the ventral (or anterior) plane of the radio-capitellar joint. Both the affected and non-affected limbs were examined. Three clinicians carried out the POCUS examination: two orthopaedic surgeons and an orthopaedic resident in training, with a view to identifying PFP and SFE. Confirmation of diagnosis was after successful reduction which was achieved with or without the classic click and with painless movement of the affected limb within 15 minutes of reduction. Positive fat pad sign on POCUS with or without pain/movement restriction of the affected limb were X-rayed and the limb was put into a cast or brace to immobilize it. These patients were later reviewed at 3-5 days, with further immobilization and repeated X-ray at 3 weeks post-injury if they were experiencing ongoing symptoms. Of the 205 patients, 196 were diagnosed with pulled elbow; 9 patients had fractures. Of the patients with pulled elbow, 156 demonstrated positive SFE on POCUS. To diagnose pulled elbow, the sensitivity of SFE was 79.5%, and the specificity was 100%. The presence of a positive fat pad with no SFE was both 100% sensitive and specific for fracture diagnosis. This was a larger, prospective study including clinical diagnosis of pulled elbow and therefore included patients with fractures who mimicked a pulled elbow. In addition to identifying signs to suggest pulled elbow it also looked for signs to suggest fractures. Patients were scanned by members of the orthopaedic team with no description of the level of POCUS experience, however, the images were saved and reviewed by a blinded radiologist. One patient in this study had a fat pad sign with pulled elbow; this child was a late presentation where the elbow had not been reduced for over 24 hours. The presence of this was considered secondary to delayed treatment, allowing for intra-articular fluid to accumulate which is in keeping with the experiences of the authors of this review.

However, Rabiner et al. [15] found no definitive link between PFP sign and delayed presentation.

### Partial Eclipse Sign

Tsai and Chiang [17] wanted to determine the etiology and pathophysiology of pulled elbow in their prospective case series. These included 13 patients considered to have pulled elbow who were reviewed and scanned by 1 orthopaedic surgeon in an orthopaedic clinic from June to October 2022. POCUS was performed in the transverse view at the level of the radial head. ‘Partial eclipse sign' on POCUS was diagnostic for posterior synovial fringe entrapment between the radial head and annular ligament. All 13 patients demonstrated partial eclipse sign which resolved post-reduction. Longitudinal view on POCUS also detected impingement of the posterior synovial fringe between the annular ligament and radial head. Images were reviewed by a musculoskeletal sonographer blinded to any patient details. Tsai and Chiang

[17] concluded that POCUS could be used to diagnose pulled elbow and prevent unnecessary elbow reduction in cases that mimic pulled elbow. They also concluded that pulled elbows are caused by posterior synovial fringe dislocation. This supported previous studies advocating for the identification of SFE. Given the small sample size, data could not be generalized which limits the power of the study. Inter-observer agreement was discussed, with near perfect agreement of the eclipse sign. This study referenced the presence of the J- sign but postulates that partial eclipse sign is better detected.

## Conclusion

POCUS is a valuable imaging modality for diagnosing pulled elbow due to its bedside accessibility and safety for paediatric patients. Many, albeit limited and small, studies have demonstrated POCUS to be reliable in the diagnosis of pulled elbow and in confirming successful reduction. The value of POCUS is greatest in cases where history is lacking or ambiguous. Children who have a positive hook sign or J- sign and absence of fat pad sign on POCUS can be managed as pulled elbow and undergo reduction. In cases where a fat pad sign is present and no positive features for pulled elbow exist, bony injuries should be considered and X-rays should be taken. Additional prospective studies of children presenting with elbow injury would be required to accurately determine the diagnostic characteristics of POCUS in pulled elbow.
